# Recent Advances on Thermoelectric Silicon for Low-Temperature Applications

**DOI:** 10.3390/ma15031214

**Published:** 2022-02-06

**Authors:** Dario Narducci, Federico Giulio

**Affiliations:** Department Materials Science, University of Milano Bicocca, v. R. Cozzi 55, I-20125 Milan, Italy; f.giulio1@campus.unimib.it

**Keywords:** thermoelectricity, silicon, energy filtering, heat harvesting, Internet of Things

## Abstract

Silicon is the most widely used functional material, as it is geo-abundant and atoxic. Unfortunately, its efficiency as a thermoelectric material is very poor. In this paper, we present and discuss advances of research on silicon and related materials for thermoelectric applications, mostly focusing on the comparison between the two strategies deployed to increase its performance, namely either reducing its thermal conductivity or, in polycrystalline materials, increasing its power factor. Special attention will be paid to recent results concerning silicon thin films. The enhancement of Si performances has motivated efforts to develop integrated heat microharvesters operating around room temperature, which will be reviewed also in view of their applications to power wireless sensors for the Internet of Things.

## 1. Introduction

Thermoelectricity has just celebrated its 200th anniversary. Since the official date of its discovery by Seebeck (1821), thermoelectric phenomena have found extensive applications in metrology, to measure temperatures, and refrigeration, using Peltier cells. Heat harvesting, although attractive, has found instead much more limited exploitations. Save for their use in radioisotope thermoelectric generators (RTGs) to power deep-space probes, the efficiency of thermoelectric generators (TEGs) was for a long time too small to provide a viable tool to convert heat into electric energy, so that TEGs could find terrestrial uses only when alternate ways to make electric power available were impossible.

As is well known, the basic problem with thermoelectricity is that the device efficiency scales with a dimensionless quantity, named thermoelectric figure of merit (ZT), which depends on the materials electrical conductivity σ, its thermal conductivity κ, and its Seebeck coefficient α as $ZT=(\sigma \alpha^{2}/\kappa )T$, where *T* is the absolute temperature and the thermal conductivity is the sum of electronic and lattice contributions. Quite unfortunately, no material displays large σ along with small κ values. Additionally, σ and α report opposite trends with carrier density, with σ increasing and α decreasing when the carrier density increases. Thus, despite the effort to select electrically conductive materials with small lattice thermal conductivity, for almost a century, no material could be obtained with ZT greater than 1 [1]. Nanotechnology could largely break this deadlock, providing ways to more independently control σ and κ. As a result, over the last two decades, ZT could jump up by almost a factor three, revamping scientific and technological interest toward TEGs and thermoelectric coolers (TECs).

As for any heat engine, also for TEGs, the maximum efficiency scales with $1-T_{L}/T_{H}$, where $T_{H}$ and $T_{L}$ are the temperatures of the hot and cold sinks. Over temperature differences such that the dependency of *Z* on *T* may be neglected, one may write

(1) $$\eta =\frac{T_{H}-T_{L}}{T_{H}}\frac{\sqrt{1+Z\bar{T}}-1}{\sqrt{1+Z\bar{T}}+T_{L}/T_{H}}$$

where $\bar{T}=\left(T_{H}+T_{L}\right)/2$. Therefore, for any given ZT, larger efficiencies may be achieved by exchanging heat at higher temperatures. This was the celebrated case of SiGe alloys used in RTGs, which may operate with $T_{H}\approx 783$ K against a cold sink typically at $T_{L}\approx 450$ K [2].

However, efficiency is only one part of the problem. In standard heat engines, high efficiencies are relevant, since heat has a cost. When instead heat is recovered at no cost (waste heat), η is only one factor entering the overall evaluation of TEG profitability. Capital costs and output power densities, along with power marginal utility, also need to be considered. Furthermore, over the last few years, growing attention has been paid to the geo-availability of the raw materials upon which TEGs are based. While Bi${}_{2}$Te${}_{3}$ and its alloys are nowadays a standard for heat harvesting around room temperature, the limited abundance of tellurium (comparable to that of platinum) is constraining bulk applications of Bi${}_{2}$Te${}_{3}$-based TEGs. This, along with toxicity issues of other once-popular thermoelectric materials (e.g., PbTe), has readdressed research toward novel classes of materials. Among them, polymers have been largely considered as key competitors, since they are easy to prepare and to process, have low costs, and are non-toxic. However, despite the significant improvements recently reported [3], still, their ZT remains too low. Better results were instead reported with polymer-based composites, wherein either organic or inorganic fillers contribute to raise the efficiency [4]. Quite remarkably, carbon nanotubes embedded in a poly(3,4-ethylenedioxytiophene):tosylate matrix led to a power factor of 1.2 mW K${}^{-2}$m${}^{-1}$ and a ZT of 0.4 at room temperature [5], approaching the threshold for practical usability. Furthermore, the chemical modification of composites by using ionic liquids was reported to enable ZT of 0.70 [6]. Several inorganic materials have been also considered to replace tellurides at low temperatures. Among them, Mg${}_{2}$Si, CoSi, and SrSi${}_{2}$ were shown to qualify for low-temperature applications [1,7], with ZT of 0.50, 0.35, and 0.4, respectively, at ≈300 K.

A rather special place in this search for low-temperature thermoelectric materials is played by silicon, its alloys, and its (nano)composites, due to the large abundance of Si, its non-toxicity, and its easy electronic integrability, which is especially relevant for low-temperature applications.

The aim of this paper is to present and discuss recent advances of research on silicon and related materials for thermoelectric applications. Silicon (mostly in the form of nanoparticle pellets) has been the subject of many investigations in the 1990s for high-temperature applications. Although these studies will be reviewed in Section 2, the main interest of this mini-review is about low-temperature applications, which are emerging as a novel area of exploitation. Differently from high-temperature conversion, low-temperature heat harvesting fully enables the use of thin films, where silicon integrability sets it apart from most other materials. Since integrated structures can sustain only modest temperature differences, Si-based TEGs mostly qualify for microharvesting applications, which will be shown to be a key enabling technology for applications ranging from the Internet of Things (IoT) to healthcare support and monitoring.

We will mainly compare two strategies that have been considered to improve Si thermoelectric efficiency. A more general presentation of progresses on thermoelectric silicon was covered by one of the present authors in two previous reviews [1,8]. Additional excellent reviews were written by Schierning [9], Perez et al. [10], and Gadea et al. [11]. Furthermore, we will specifically address recent results concerning bulk silicon—including thin films. Readers interested in silicon nanowires (SiNWs) may refer to specialized reviews recently published on this class of systems [12,13].

This paper is organized as follows. After a brief overview on silicon and its thermoelectric properties, also covering first advances to qualify silicon for thermoelectric applications (Section 2), recent progress will be reviewed, following the two lines of attack of reducing its thermal conductivity in single crystals (Section 3) and to increase its power factor in poly/nanocrystalline silicon (Section 4). Materials achievements have disclosed novel opportunities of applications, which will be then presented and discussed in Section 5. Although most mature applications have targeted high-temperature heat harvesting, the availability of thermoelectrically efficient Si thin films shifts attention to novel low-temperature applications, where the material qualifies for both micro-harvesting and localized cooling. An outlook to further materials developments and to applicative contexts will close the paper.

## 2. Silicon: From ZT≈0.01 to Its Practical Usability

Silicon is the most widely used and better known functional material, with a fantastic technology supporting its use, which today ranges from microelectronics to photovoltaics. Unfortunately, its efficiency as a thermoelectric material is rather poor.

The thermoelectric properties of single-crystalline silicon were reported in the 1950s by Geballe and Hull [14], and more recently re-measured by Fulkerson et al. [15] and Stranz et al. [16]. As known, Si has a high power factor (PF) $\sigma \alpha^{2}$ at high doping levels (>10${}^{18}$ cm${}^{-3}$) [8]. However, due to its high thermal conductivity (≈140 W/mK at room temperature), its ZT is ≈0.01 at room temperature, despite a PF of about 5 mW/mK${}^{2}$.

Attempts to increase Si thermoelectric efficiency have been mostly pursued by decreasing its thermal conductivity (without reducing its electrical conductivity). Alloying provided a successful strategy in this sense. The first studies on SiGe alloys were carried out in 1964 [17], obtaining a maximum ZT of ≈1 and 0.7 at 1100 K for n- and p-type Si${}_{0.70}$Ge${}_{0.30}$ alloys. The synergistic effect of alloying and polycrystallinity on κ was explored by Rowe et al. [18] and Vining et al. [19]. Further reduction of the thermal conductivity by compressing (sub)micrometric powders of n-type Si${}_{0.8}$Ge${}_{0.2}$ were achieved, although at the cost of an increased electrical resistivity [19].

Despite the already mentioned use of SiGe alloys in radioisotope thermoelectric generators [20], the efficiency of Si and SiGe alloys did not progress appreciably for about four decades, from 1960 to 2000. It was just with nanotechnology that this state of affair could change. Dimensional constraints were shown to be effective at reducing κ. The damping of thermal conductivity by incoherent phonon scattering at SiNW walls was found capable of reducing Si thermal conductivity by almost two orders of magnitude at room temperature. SiNWs with diameters smaller than 100 nm [21,22] provided ZT values close to 1. Comparable results were reported also by nanolayers (i.e., thin films with thickness below 200 nm) [23]. Nanotechnologic control of the thermal conductivity was also achieved in bulk silicon. Very high ZT values at 1173 K were reported in hot-pressed Si${}_{0.8}$Ge${}_{0.2}$ nanopowders with grain sizes of 10–20 nm, reaching ZT=1.3 in P-doped pellets and ZT=0.95 in B-doped pellets [24].

It should be noted that raising ZT either by decreasing κ or by increasing the power factor is in no way equivalent. While they both concur at defining efficiency, only PF sets the power density $\dot{w}$ delivered by a TEG with a leg length *ℓ* over a given temperature difference ΔT [25]:

(2) $$\dot{w}=\frac{\alpha^{2}\sigma }{4\ell }{\left(\Delta T\right)}^{2}.$$

Thus, neglecting thermal contact resistances, when the heat flux $\dot{q}$ is given, then $\Delta T\propto \dot{q}/\kappa$, so that a small thermal conductivity sustains a larger temperature difference—and therefore a larger $\dot{w}$. Instead, when the TEG operates between two thermostats, the opposite is true, namely the input heat flux scales with κΔT, so that a TEG with a vanishing thermal conductivity admits no heat, correspondingly yielding no power output [26]. Real-world operative conditions for TEGs are usually in between these two extremes. In any case, especially for harvesters converting waste heat (e.g., microharvesters, cf. Section 5), maximizing PF is often more convenient than minimizing the thermal conductivity [27]. Thus, materials with a relatively large κ (anyway <10 W/mK) but with large power factors may be of interest for waste heat harvesting.

Research on silicon thin films for thermoelectric applications around room temperature has moved along both directions to improve ZT, namely reducing Si thermal conductivity while retaining its PF and increasing PFs in polycrystalline films, where κ is reduced by phonon scattering at grain boundaries.

## 3. Decreasing Thermal Conductivity in Single-Crystalline Silicon

On the first route, alloying and defect engineering (including impurities and dopants) have been investigated. Germanium has been considered, extending to thin films the strategy successfully deployed in ingots and pellets despite the cost and the limited geo-availability of the element. Not surprisingly, the use of SiGe alloys was confirmed to be successful also in thin films, improving efficiency up to ≈7% [28]. On defect engineering, computational analyses [29] reported how at dopant concentrations of $5\times 10^{20}$ cm${}^{-3}$, thermal conductivity at 300 K falls from 137 W/mK (undoped Si) down to 18, 39, and 57 W/mK in As-, B-, and P-doped silicon. Dopants mostly reduce κ due to mass disorder effect, with bond disorder contributing only for P doping. No significant effect is instead ascribed to lattice strain.

The wise use of structural defects was exploited by Bennett et al. in a series of papers dealing with single-crystalline Si (scSi) thin films. Si${}^{+}$ ion implantation in a 100 nm thick scSi film (P-doped, $10^{19}$ cm${}^{-3}$) injected a very large number of vacancies (density $\approx 8\times 10^{19}$ cm${}^{-3}$), yet not fully amorphizing the film. A subsequent rapid thermal annealing reported that after 10 s at 600 ${}^{\circ }$C, an optimal compromise between the reduction of κ and recovery of σ could be reached. This result could be explained in view of the fact that an optimal vacancy density exists at which its effect on carrier mobility is comparable to that of ionized impurities, thus not impacting the electrical conductivity although hindering phonon diffusion. Then, a 20-fold reduction in thermal conductivity was found, while the electrical conductivity and Seebeck coefficient (and PF thereof) could be almost fully retained. Then, a ZT of 0.16 at 300 K was obtained, with the power factor being 3.5 mW/mK${}^{2}$, which is fully comparable to that of standard scSi [30].

Dislocations were also used to tailor thermal conductivity and the power factor [31]. However, in this case, the large density of dislocation generated by ion implantation does not allow to recover the pristine values of electrical conductivity. Quite interestingly, extended annealing led to a simultaneous increase of α and σ, bringing the PF to 6.6 mW/mK${}^{2}$. Energy filtering (cf. Section 4) was speculated to be a possible cause. No in-plane thermal conductivity was reported, so that no ZT could be computed. However, a cross-plane κ down to 50 W/mK was observed.

A further reduction of κ could be attained in nanocrystalline silicon (ncSi). The effect of reduced grain size was studied in Si films deposited by Low-Pressure Chemical Vapor Deposition (LPCVD) at 610 ${}^{\circ }$C onto either oxidized Si or quartz substrates, then boron-implanted to a nominal doping level of $3\times 10^{19}$ cm${}^{-3}$, and finally annealed at 1050 ${}^{\circ }$C for 30 min in N${}_{2}$ [32]. The average grain size was found to range between 50 and 112 nm, increasing with film thickness. This caused κ to drop down to about 45 W/mK. The interdependency of α on σ fitted instead the standard Pisarenko’s plot, although the carrier mobility at room temperature was as large as 92 cm${}^{2}$/Vs.

## 4. Increasing PF in Polycrystalline Silicon

On the second route, attempts to increase the PF of polycrystalline silicon were also reported.

In poly/nanocrystalline silicon (with already low thermal conductivity), one may attempt to increase the PF by a host of methods, including energy filtering and modulation doping. Manifestly enough, the simple introduction of grain boundaries (GBs) is expected to lead to a degradation of both thermal and electrical conductivities, with little or no ZT improvement. This is true and widely confirmed in non-degenerate polycrystalline silicon, namely for doping levels up to $10^{18}$ cm${}^{-3}$ [33]. Instead, this is not necessarily the case in nanostructured systems. As an example, modulation doping was found to be beneficial in mixtures of two types of SiGe nanoparticles, one of which heavily doped. A major improvement of PF due to a mobility enhancement was reported [34], with the mobility increasing up to 50% in (Si${}_{95}$Ge${}_{5}$)${}_{0.65}$(Si${}_{70}$Ge${}_{30}$P${}_{3}$)${}_{0.35}$ mixtures. Then, a ZT≃1.3 at 900 ${}^{\circ }$C could be achieved.

Extended investigations of the usability of energy filtering to improve PF in ncSi were also reported. Energy filtering, namely energy-selective scattering of charge carriers, was suggested as a tool to improve PFs in the 1990s [35] and was the subject of major theoretical efforts [36,37,38,39,40,41,42,43,44] since. Its occurrence was experimentally exploited in superlattices [45,46,47], silicides [48], chalchogenides [49,50,51,52,53], nanocomposites [4,54,55,56,57], and halogenides [58] as well. Its impact on thermoelectrics has been recently reviewed by Gayner and Amouyal [59].

**Figure 1:**
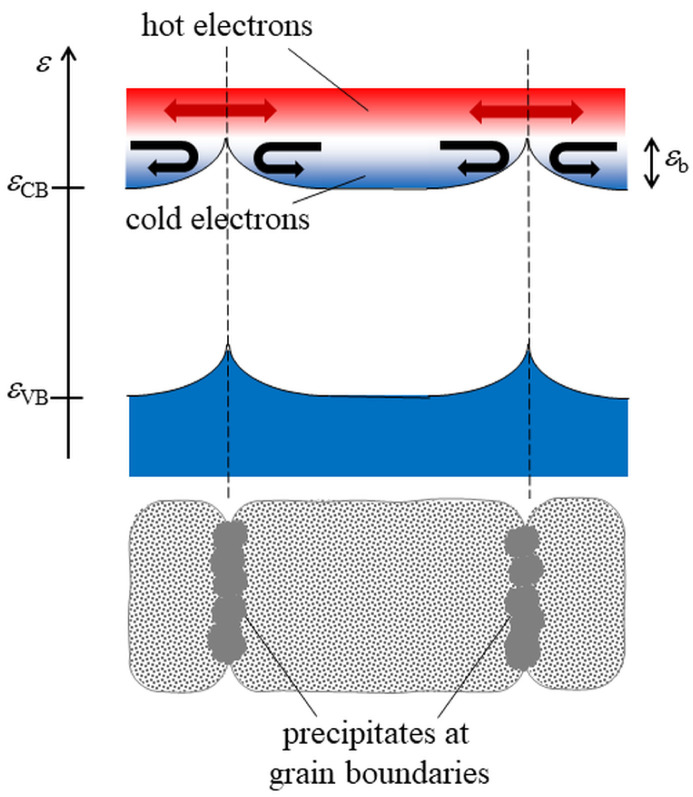
Schematics of the energy filtering of electrons. For barriers of height $\epsilon_{b}$, electrons with kinetic energy $\epsilon -\epsilon_{\mathrm{CB}}>\epsilon_{b}$ travel across grains boundaries without being scattered, while ‘cold’ electrons are trapped within barrier pairs. ‘Hot’ (mobile) carrier density decreases but their mobility increases, causing σ not to change significantly. At the same time, the Seebeck coefficient increases—therefore improving the PF. Note that energy filtering is effective only when barrier spacing is smaller than the carrier mean-free path, preventing their thermalization within the grain.

In a nutshell, in single crystals, charge carriers are fully thermalized. Then, electrons (holes) diffuse and/or drift with an energy of about $k_{B}T$ (where $k_{B}$ is the Boltzmann’s constant) above the minimum of the conduction band $\epsilon_{\mathrm{CB}}$ (below the maximum of the valence band $\epsilon_{\mathrm{VB}}$). When barriers of height $\epsilon_{b}$ build up, typically at grain boundaries, they scatter all electrons (holes) with an energy smaller (larger) than $\epsilon_{b}$ ($-\epsilon_{b}$), save for those overcoming the barrier by thermionic emission [60]. This is the standard mechanism of grain boundary scattering, occurring when barriers are large. However, for conveniently small $\epsilon_{b}$, barriers select carriers upon their kinetic energy, enabling hot carriers, namely electrons (holes) with energy $\epsilon >\epsilon_{\mathrm{CB}}$ ($\epsilon <\epsilon_{\mathrm{VB}}$), to move across grain boundaries without being scattered (Figure 1)—namely filtering carriers upon their energy. Mobile carrier density decreases, because part of the charge carriers are localized within grains. However, mobile (hot) carriers display a larger mobility, which may mitigate, compensate, or even overcompensate the decrease of carrier density. At the same time, the decrease of carrier density increases |α|, therefore causing the PF to significantly increase. For energy filtering to be effective, two conditions must be met, namely $|\epsilon_{b}|$ must be ≈$k_{B}T$ to enable a proper compromise between carrier density decrease and mobility enhancement, and barrier spacing must be smaller than the carrier mean-free path to prevent their thermalization within the grains.

### 4.1. Silicon Thin Films

High doping levels in silicon were studied by Jugdersuren et al. [61], who deposited Si film by LPCVD, setting the temperature of the substrate to 250 ${}^{\circ }$C. Boron was ion implanted, and the specimen were then annealed up to 700 ${}^{\circ }$C. All samples displayed a columnar microstructure, possibly with an amorphous incubation layer. Depending on sample post-deposition annealing, the in-plane grain size ranged from 5 to 12 nm. Thermal conductivity as low as 0.76 W/mK was reported. Electrical conductivity displayed almost constant values from 70 to 300 K, increasing up to 120 $\Omega^{-1}$ cm${}^{-1}$ at the highest nominal doping level ($3\times 10^{21}$ cm${}^{-3}$), while the Seebeck coefficient reported a roughly linear increase with *T*. The largest power factor at 300 K was 0.2 mW/mK${}^{2}$, which was almost identical for any doping level above $10^{21}$ cm${}^{-3}$. Figures of merit of 0.1 at 300 K, i.e., ten times larger than in scSi, could be obtained.

SiGe alloys were also considered [62]. Thin films of Si${}_{74}$Ge${}_{26}$ alloys were grown by LPCVD on a Si (100) wafer that was previously coated with a SiO${}_{x}$ film and an amorphous Si thin layer. Upon ion implantation followed by rapid thermal annealing, the film was submitted to additional annealing at 1000 ${}^{\circ }$C for one hour. The film developed columnar grains with an average in-plane grain size of ≈275 nm. The combined effect of alloying and polycrystallinity resulted in an in-plane thermal conductivity of 2.2 W/mK at 300 K that, along with a PF of ≈1.5 mW/mK${}^{2}$, led to an excellent ZT of 0.2 at room temperature.

In a more recent study mostly focused on ternary Si${}_{1-x-y}$Ge${}_{x}$Sn${}_{y}$ alloys [63,64], Si${}_{1-x}$Ge${}_{x}$ control samples doped with boron (≈$10^{20}$ cm${}^{-3}$) were also prepared and analyzed. Smaller grain sizes were obtained (≈10 nm). Rapid thermal annealing only (15 s at temperatures up to 1150 ${}^{\circ }$C) was carried out. Thus, only a very small improvement of the PF was reported (up to 0.1 mW K${}^{-2}$m${}^{-1}$).

The occurrence of energy filtering in Si thin films has been also reported. Possibly, the first evidence of this phenomenon in silicon dates back to 1988, when Vining reported that prolonged annealing of bulk boron-doped ncSi ingots with carrier density exceeding $10^{19}$ cm${}^{-3}$ caused an unexpected improvement of the power factor along with an increase of hole mobility, up to 40 cm${}^{2}$/Vs at 300 K [19,65]. More specifically, the Seebeck coefficient was found to increase with the boron content between 1 and 20%, which is a very uncommon trend violating Pisarenko’s relation. As SiB${}_{3}$ precipitates were detected, a second phase with larger α was conjectured to be responsible for the enhanced thermopower, although by itself, this could not explain mobility vs. carrier density data. Although energy filtering was not taken as a possible explanation, Vining’s findings were congruent with an early report by Seager [66] who also had reported about an increase of hole mobility “approaching (within, say factors of 2 or 3) single-crystal values” in heavily boron-doped ncSi.

More recently, an unexpected concurrent increase of the Seebeck coefficient and of the electrical conductivity was observed in heavily boron-doped nanocrystalline silicon films upon annealing at temperatures ≥800 °C [67]. The unusual, concurrent increase of the power factor (PF) was related to the precipitation of a boron-rich phase at GBs [68], setting a double potential barrier that filters charge carriers. It is essential to stress that denuded grain boundaries in Si set a too high barrier for energy filtering to be beneficial [60]. Thus, boundary decoration (by boron or possibly other species) is apparently mandatory to observe an increase of the PF. In addition, as already noted, an increase of the PF requires the grain size to be small compared to the carrier mean-free path, letting holes move in a semi-ballistic regime [67,69]. If such a scenario is met, only hot carriers diffuse upon the application of a thermal gradient. As a result, mobile carriers move through the film over the barriers, then as if no grain boundary were present. The concurrent increase of Seebeck coefficient and electrical conductivity was confirmed by the temperature dependence of carrier mobility and density [70]. Mobility reports a single-crystal-like trend, since, as anticipated, mobile carriers overcoming the barriers are not scattered. Instead, carrier density is much less sensitive to the temperature, as expected in degenerate silicon. However, carrier density decreases upon annealing above 800 ${}^{\circ }$C, consistently with the filtering mechanism [71]. Energy filtering in silicon was further corroborated by theoretical modeling and computational simulations [72,73]. Very recently, additional evidence was shared about the (adverse) role that hydrogen plays in ncSi grown by chemical vapor deposition using silane as a precursor [74]. Hydrogen was found to interfere with boron precipitation, reducing the portion of grain boundaries where energy barriers establish. Although a detailed mechanism for hydrogen interference could not be demonstrated, full hydrogen removal led to even higher PFs, above 30 mW/mK${}^{2}$.

Comparison among literature findings seemingly provides guidelines to enable energy filtering in nanocrystalline Si. Three requisites should be met: (a) free dopant concentrations must exceed their solubility threshold at the annealing temperature to allow the dopant to precipitate at grain boundaries; (b) annealing conditions must be such that dopant diffusion lengths are at least comparable to the grain size, enabling excess dopant to diffuse to grain boundaries; and (c) the grain size must be smaller than the carrier mean-free path to prevent carrier thermalization.

### 4.2. Silicon Nanocomposites

In recent years, a number of investigations have addressed nanocomposites of Si nanocrystals embedded in a hydrogenated amorphous silicon (aSi:H) tissue, which are often referred to (quite misleadingly) as ‘microcrystalline silicon’ (μc-Si:H). In a series of papers, Loureiro et al. [75,76] reported about the effect of radio-frequency power density used to grow in situ boron-doped films, reporting about an anomalous increase of the electrical conductivity at a constant Seebeck coefficient, which was tentatively related to the occurrence of energy filtering due to boron precipitation at grain boundaries. However, due to the prevailing carrier scattering in the amorphous matrix, PF could not exceed 0.4 mW K${}^{-2}$m${}^{-1}$. Comparable results were obtained by Acosta et al. [77], who also analyzed in detail the role played by hydrogen in their system. However, also in this case, the best PF was limited by the presence of the amorphous tissue that, while positively impacting ZT, limited the PF to ≈0.2 mW K${}^{-2}$m${}^{-1}$.

The effect of annealing on heavily boron-doped Si nanocrystals embedded in amorphous hydrogenated Si (aSi:H) was reported by Zhang et al. [78]. Annealing at 600 ${}^{\circ }$C for 10 min of samples with a nominal B density ≥$10^{20}$ cm${}^{-3}$ led to the full recrystallization of the thin film and to the formation of grains with sizes smaller than 5 nm. In such systems, violations of the standard Pisarenko relation were observed and tentatively explained as a result of energy filtering. Rather unfortunately, no annealing above 600 ${}^{\circ }$C and/or for longer times were attempted. Therefore, the largest PF was of ≈2 mW K${}^{-2}$m${}^{-1}$, yet there was a remarkably large value in nanocrystalline films.

In addition to μc-Si, also other nanocomposites have recently attracted interest as thermoelectric materials because of their potential to cause a reduction of the thermal conductivity although saving acceptable PFs.

Sintered oxidized Si nanoparticles and nanocrystalline Si embedding SiO${}_{x}$ nanoparticles were studied by Petermann et al. [79]. Doped silicon nanoparticles of size of ≈15 nm and covered by an oxide layer formed by exposure to air for variable times were processed by current-assisted sintering, yielding grains ranging between 60 and 101 nm, decreasing with increasing oxygen exposure times. Due to their pristine size, oxidized nanoparticles caused a large amount of oxygen to be incorporated into the pellets, from 9.5 to 25.5 wt %. Thermoelectric transport properties were analyzed. They showed that both electron mobility and thermal conductivity decrease with increasing silicon oxide content, with PF falling from 3 to 1.1 mW/mK${}^{2}$ at 950 ${}^{\circ }$C. Nonetheless, due to the lower thermal conductivity (decreased down to 9 W/mK at room temperature), a maximum figure of merit of 0.45, still at 950 ${}^{\circ }$C, was obtained when the silicon oxide mass fraction was either 9.5 or 21.4%.

The effect of direct addition of SiO${}_{x}$ to ncSi thin films was extensively studied by Shiomi [80]. Adopting a multi-scale computational approach, it was shown how while the thermal conductivity of ncSi is sensitive to the average grain size and to the contact thermal conductance at grain boundaries, it is instead insensitive to the grain-size distribution. Following this guideline, samples of a few tens of nm were obtained, which were engineered in such a way to display a contact thermal conductance in the order of $10^{8}$ W/m${}^{2}$K so as to design a material with a resulting thermal conductivity ≪10 W/mK. To this end, test structures were made by bonding two (100) single-crystalline Si film with an interposed oxide layer with variable thickness. Annealing at temperatures <1100 ${}^{\circ }$C developed oxide nanoparticles modulating κ at the Si–Si interface. Notably, the SiO${}_{x}$ nanoparticles were crystalline, retaining a Si structure despite an oxygen content exceeding its solubility in silicon. Contact conductance was measured to range between $10^{8}$ and $10^{9}$ W/m${}^{2}$K. On such premises, Si nanoparticles with a diameter of 6 nm were exposed to air to form a native oxide layer. Then, such core–shell particles were plasma-sintered. The power factor was limited (increasing from 0.5 mW/mK${}^{2}$ at room temperature to 2.5 mW/mK${}^{2}$ at 850 ${}^{\circ }$C), but the very low κ led to a remarkable ZT of ≈0.6 at 850 ${}^{\circ }$C.

## 5. Low-Temperature Applications

Despite its standard limited performances, still, silicon has found applications in microharvesters where its integrability has prevailed over its fair efficiency [81,82,83,84]. The availability of low-cost, integrated microTEGs is largely considered a key enabling technology supporting the development of the Internet of Things (IoT). As known, in a network of intercommunicating sensors (and possibly low-power actuators), nodes exchange information using wireless supports, either short-ranged (e.g., bluetooth) or long-ranged (e.g., WiFi). Still, sensing nodes require powering. On-grid sensors cover only a small faction of the grand total. Even when an electric line is within reach, installation costs may discourage the use of the grid, which is instead impossible for remote/distributed sensing. Batteries are seemingly handy solutions. Yet, maintenance costs (i.e., labor cost required to periodically replace exhausted batteries) suggest the use of renewable power sources, delivering powers in the order of milliwatts. TEGs, both bulk and integrated, already qualify for this range of power (along with photovoltaic minicells and, for some applications, vibrational harvesters) [85,86].

However, low-temperature applications are not limited to IoT. As noted [87], integrated harvesters powering flexible electronic devices are largely needed for healthcare, both as portable monitoring devices embedded in garments or, possibly, directly integrated in the patients’ body. In this sense, the availability of silicon nanomaterials (Si nanotubes) integrated in fabrics [88] or smart designs using peel-off technologies to transfer harvesters and Si-based electronics onto flexible supports [87] open challenging and relevant technological perspectives.

MicroTEGs commonly adopt two types of layouts. In a *parallel layout*, heat flows parallel to the substrate with the thermoelectric legs thermally insulated from the substrate. In a *normal layout*, instead, heat flows normal to the substrate with cavities underneath the legs preventing thermal shunts from the substrate [82,89]. The parallel layout is ideally compatible with planar technologies, but its performances are limited by the residual thermal shunt due to the membrane the legs sit on. Furthermore, their mechanical stability (fragility) is an issue. Normal layouts are instead more robust, but they severely waste the available temperature difference between hot and cold heat sinks. Interesting examples of smart solutions reported the use of bulk silicon frameworks with Si nanowires grown across facing sides of a supporting Si frame [90,91]; and, for normal layouts, thinned substrates minimizing series thermal resistance were also demonstrated [92].

Both types of layout have displayed significant improvement of their performances over the last twenty years, with power densities up to 12.3 μW/cm${}^{2}$ over a temperature difference of 31 K [93].

Integrated TEGs using Si were computed also to meet profitability due to the scalability and to the reduced complexity of the fabrication process [89]. Although a precise computation of manufacturing costs for integrated devices is obviously impossible, it was estimated that for microTEGs obtained through standard planar manufacturing processes, the cost of fabrication is set by three factors, namely the number of required lithographic steps, the yield of the process, and the testing cost. Furthermore, cost per device depends on the number of dies per processed wafer. Materials costs, instead, have a negligible impact on the final TEG costs. Inheriting the cost model from micro-electromechanical systems (MEMSs) and considering that in general terms, microTEGs require no more than five masking steps, costs were estimated from 2.61 to 4.43 USD per die, taking square dies of 20×20 mm${}^{2}$. Adding packaging costs, the predicted power cost was computed to be between 60 and 120 USD/W. Although the figures may look discouraging, it was noted that the cost structure of microTEGs is independent of that of dissipaters, as their contribution to the final power cost is shown to be negligible. Furthermore, for the larger production volumes expected by the expanding IoT market, production costs might largely scale down, which is usual for integrated electronic devices. However, even at current estimated costs, microTEGs already provide a profitable way to power off-grid sensing nodes. This follows considering that for an exemplar 1 mW node with a lifetime of 1000 days, the *energy* needed to power it sums to $6.24\times 10^{5}$ J. At a current battery energy cost of 150 USD/MJ [89], powering with batteries costs 9.36 USD (neglecting maintenance) while converting waste (free) heat with microTEGs sets the cost to 0.04–0.12 USD. This major difference simply depends on the fact that waste heat is free, so only capital costs enter the computation. Considering battery maintenance (labor) costs, the convenience of microTEG over batteries becomes even larger.

Prototypes anticipating this path have been recently reviewed by Yan [82] and Jaziri et al. [94]. Important advances on the route of making integrated TEGs fully compatible with standard microelectronic technologies were presented by Yang et al. [84,95]. Differently from most previous implementations [81,93], in this case, the device was designed adapting a well-established BiCMOS (Bipolar Complementary Metal-Oxide-Semiconductor) layout. Legs (45×2μm${}^{2}$) were made of polycrystalline Si and SiGe of unspecified doping over a total device area of 1.2×1.2 mm${}^{2}$. A series leg connection led to a voltage factor of 23.53 V/Kcm${}^{2}$, enabling a thermovoltage >3 V over a temperature drop of 10 K. It was stressed how high output voltages are crucial to microTEG usability in integrated devices, which is even more significant than the power density itself that, after packaging, amounted to 9.7 μW/cm${}^{2}$. Performances were found comparable to those achieved using CMOS-incompatible processes making use of BiSbTe/BiSb legs. Instead, voltage factors outperformed those attained with previously fabricated CMOS-compatible Si-based integrated heat harvesters, confirming how Si already qualifies for IoT applications, being fully competitive with tellurides.

## 6. Summary and Outlook

Silicon usability as a thermoelectric material is largely increasing. On the material side, recent progress has been reported, remarking on not only the renewed attention toward the material, in all its forms, but also significant improvements of its power factor and its figure of merit. In single-crystalline silicon films, the lesson learned from nanotechnology and concerning the possibility of decreasing its thermal conductivity with little harm to its power factor has been implemented in thin films with improved results. With poly/nanocrystalline thin films, which may be obtained at even lower costs, both modulation doping and energy filtering have enabled ways to enhance the power factor, leading to a consequent increase of ZT of almost two order of magnitudes, nearly aligning Si thin films with Si nanowires. As noted, several of the most significant results achieved on silicon (and on other thermoelectric materials as well) have taken advantage of theoretical and computational investigations [96,97]. Large computer-generated datasets might provide additional tools to accelerate the discovery of new performing thermoelectric materials—and of ways to modify known materials to increase their efficiency [98,99].

However, the availability of efficient and geo-abundant materials is not enough. For silicon, technologists are conceiving layouts suitable to conveniently drive heat flows also in integrated devices, both minimizing heat shunts and maximizing the temperature difference applied to the thermoelectric legs. At the same time, TEG layouts profiting from established microelectronic geometries have been demonstrated. All in all, in addition to well-established uses of silicon and silicon alloys at high temperatures, room is opening at the bottom, where silicon thin films may show potential as a thermoelectric material for low-temperature applications to provide on-board powering of microelectronic devices. It seems not too overoptimistic to expect IoT and portable healthcare devices to act as the long-awaited killer application promoting thermoelectrics, enabling TEGs to leave the limbo of niche applications and exploiting their full capability as a key enabling technology.

## Data Availability

Not applicable.

## Abbreviations

The following abbreviations are used in this manuscript:

GB	Grain boundary
IoT	Internet of Things
ncSi	Nanocrystalline silicon
RTG	Radio-isotope Thermoelectric Generator
TE	Thermoelectric
TEC	Thermoelectric cooler
TEG	Thermoelectric generator
PF	Power factor
scSi	Single-crystalline silicon
SiNW	Silicon nanowire
